# Airbag-related penetrating injuries: A case series from a level 1 trauma center

**DOI:** 10.1016/j.tcr.2023.100890

**Published:** 2023-08-08

**Authors:** Sherwan Khoschnau, Ahmed F. Ramzee, Ayman El-Menyar, Ruben Peralta, Rafael Consunji, Ahmad Kloub, Ahmed Ajaj, Husham Abdelrahman, Hassan Al-Thani

**Affiliations:** a Department of Surgery, Trauma Surgery, Hamad Medical Corporation, Doha, Qatar; b Department of Clinical Medicine, Weill Cornell Medical College, Doha, Qatar

**Keywords:** Airbag deployment, Traffic-related injury, Trauma, Penetrating, Car, Projectile material

## Abstract

**Background:**

Airbags have significantly reduced the morbidity and mortality associated with trauma following motor vehicle crashes. However, airbags can also be associated with unusual and complex patterns of injuries which could be fatal in some cases.

**Methods:**

We collected data and described a series of six cases of penetrating injuries related to airbag deployment that were treated at the Hamad Trauma Center (HTC) of Hamad Medical Corporation which is the only level 1 trauma center in the country.

**Results:**

The penetrating injuries were caused by a metal fragment from the inflator component of the airbag which acted as a projectile and was associated with two fatalities. Four of the victims were involved in head on collisions. Most injuries were directly attributable to the airbag projectile, and they occurred in vehicles that were 9 years or more since manufacture.

**Conclusion:**

This case series would help the trauma healthcare providers to better understand the airbag-related injuries which influence the management approach for road traffic injuries associated with penetrating trauma. Also, it would bring attention to injury prevention teams as well as state and industrial authorities to reevaluate safety standards in vehicles. Sharing this information with local authorities who govern product safety standards and recalls is essential to ensure that more safety actions are taken to prevent further airbag deployment injuries.

## Introduction

According to the World Health Organization (WHO), road traffic crashes are the leading cause of injuries in both low and middle-income as well as high-income countries, accounting for 1 out of 3 deaths [1]. The last few decades have seen the introduction of various measures to reduce motor vehicle related injuries including the road traffic legislations, improvement of vehicle technology, three-point seat belt as well as airbags.

Airbags were first introduced in the 1950s however, it was not generalized until the 1990s when they came into widespread use with the passage of new legislations mandating their installation in new cars [2]. The National Highway Traffic Safety administration estimates that nearly 50,000 lives have been saved by airbags since 1987 [3].

Despite the significant impact on injury mitigation, airbags themselves have been associated with unique patterns of injury. In the last two decades, there were many reports of injuries related to airbag malfunction resulting in catastrophic injuries. More recently, in 2015, multiple casualties resulted in the recall of millions of vehicles globally due to defective inflator components in the airbag resulting in explosions releasing the metal propellant canister at high force resulting in ballistic type of injuries [4].

In this case series, we present airbag inflator related injuries in six patients who were brought to the Hamad Trauma Center (HTC) in Qatar, a level 1 trauma center, that provides trauma care at no cost to the entire population of Qatar (2.9 million population). We used a convenience sample for all the admitted cases with such injuries within a specified period at the only national trauma center in the country. The Medical Research Center (MRC) of Hamad Medical Corporation (Doha , Qatar) has granted permission for this case report to be published on condition that no patient-identifiable data (including patient name and photograph) are included (IRB# MRC-04-22-719). No informed consent was needed as data were collected anonymously without direct contact with the patients.

## Case-1

**Fig. 1:**
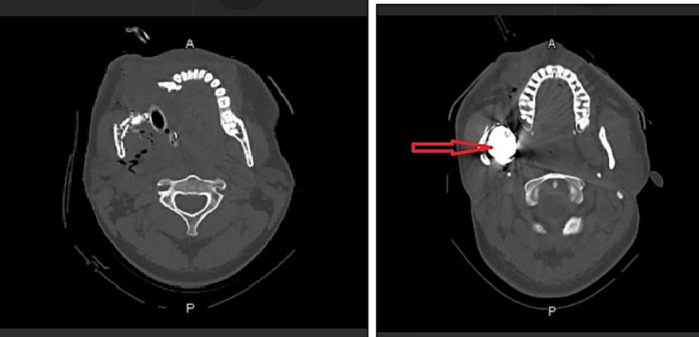
CT scan for case 1 (arrow: foreign body).

A 29-year-old male restrained driver of a 13-year-old compact sedan was injured in a head-on collision. Paramedics found him lying prone outside his car, the airbag was noted to have deployed. He had a Glasgow coma scale (GCS) of 15 and was noted to have a large laceration on the right jaw. In hospital he was conscious and alert but due to the risk of a compromised airway, he was intubated electively. He had no other obvious injuries. His head computerized tomographic (CT) scan films showed a severely comminuted fracture involving the right hemi-mandible with dislocation of the right temporomandibular joint. A 2.2 × 2.1 × 2.1 cm hyperdense foreign body (FB) was seen in the vicinity of the fracture fragment at the level of the right ramus. Multiple fractures of the anterior and lateral walls of the right zygoma and maxillary sinus reaching the floor of the right orbit were also noted (Fig. 1).

The patient was taken to the operating room (OR) and underwent exploration with retrieval of a round metallic FB and open reduction and internal fixation (ORIF) of the mandible. His post-operative course was uneventful, and he was discharged home 14 days after admission.

## Case-2

**Fig. 2:**
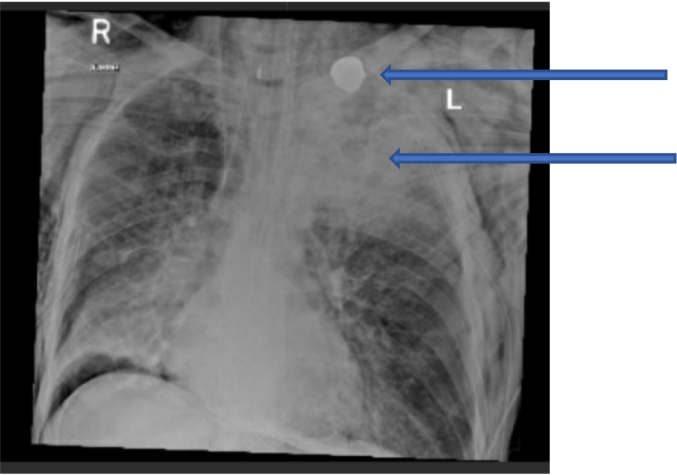
Chest X-ray revealing a foreign body in the left upper chest with significant contusions for case 2 (upper arrow: foreign body; lower arrow: lung contusion).

A 43-year-old male intoxicated restrained driver of a 3-year old sedan was injured in a frontal collision with a fixed object. There was significant damage to the vehicle and the patient was entrapped. When the paramedics arrived he had a GCS of 12, was tachycardic with a systolic blood pressure (SBP) of 90 and an oxygen saturation below 80 %. He was also noted to have a sucking wound in the left upper chest with active bleeding. The patient was intubated at the scene, and a 3-sided dressing was applied to the wound, and a needle decompression was performed. In the trauma bay, a left-sided chest tube was inserted, however his saturation did not significantly improve. A chest x-ray (CXR) showed significant lung contusions with a rounded metallic FB noticed in his upper chest and a diffuse subcutaneous emphysema with mild pneumothorax were noted (Fig. 2).

**Fig. 3:**
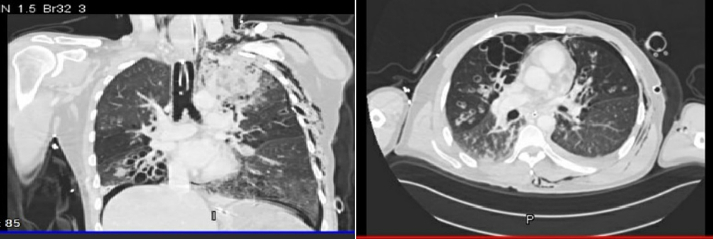
For case 2: CT scan shows pre-existing cystic bronchiectasis, extensive surgical emphysema on the left side of the chest extending to the neck and abdominal wall.

Chest CT scan showed a metallic FB lying between the left scapula and the skin. There was an evidence of pre-existing cystic bronchiectasis in both lung fields. Extensive surgical emphysema was also noted on the left side of the chest extending to the neck and abdominal wall with bilateral pneumothorax and multiple fractured ribs on the left side and manubrium sternum (Fig. 3).

The patient underwent a left thoracotomy. He was found to have a laceration in the apex of the left lung with a rounded metallic FB, bone, and cartilage fragments within the lung. Hemostasis was performed and the patient was put on extracorporeal membrane oxygenation (ECMO) due to severe respiratory failure. He expired after 4 months in the hospital while still on extracorporeal membrane oxygenation (ECMO).

## Case-3

**Fig. 4:**
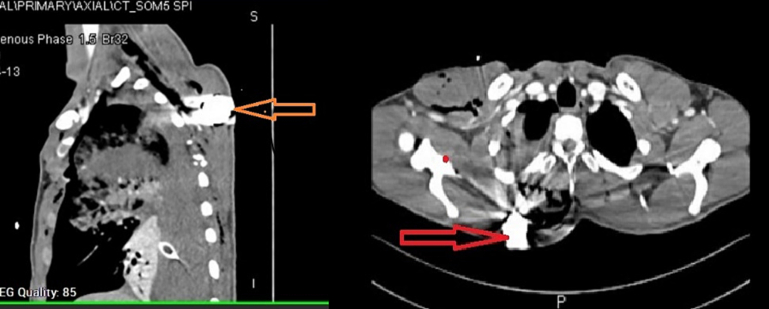
CT scan for case 3; arrows show the foreign body.

**Fig. 5:**
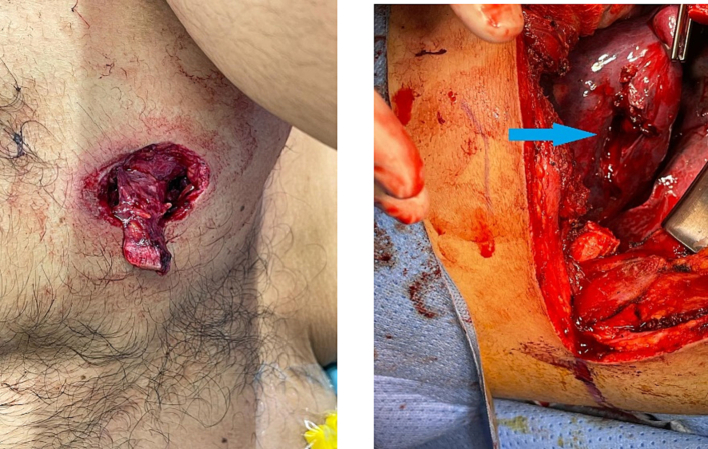
Surgical image for case 3 (arrow: the entry of the foreign body into the lung tissue).

A 24-year-old male restrained driver of an 11-year-old compact sedan was injured in a head on collision. The airbag was deployed. Paramedics found him with a GCS of 15 and a penetrating wound to the right chest. In the trauma bay he was tachypneic with diminished air entry on right side of chest. A chest tube was inserted which brought out 1 L of blood. CXR revealed right sided hemopneumothorax with a presence of a round metallic FB at the upper side of chest. The patient was intubated, and a chest CT scan was performed and revealed a dense rounded FB in the right posterior chest wall subcutaneously positioned medial to the right scapula with associated comminuted fracture of scapula with hemothorax. He was taken to the OR and a right thoracotomy was performed. A through and through laceration of the upper lobe of the lung was noted. Hemostasis was achieved and the laceration was repaired. The FB was retrieved via an incision on the back (Fig. 4, Fig. 5). The patient had an unremarkable post-operative course and was discharged home eight days post-injury.

## Case-4

**Fig. 6:**
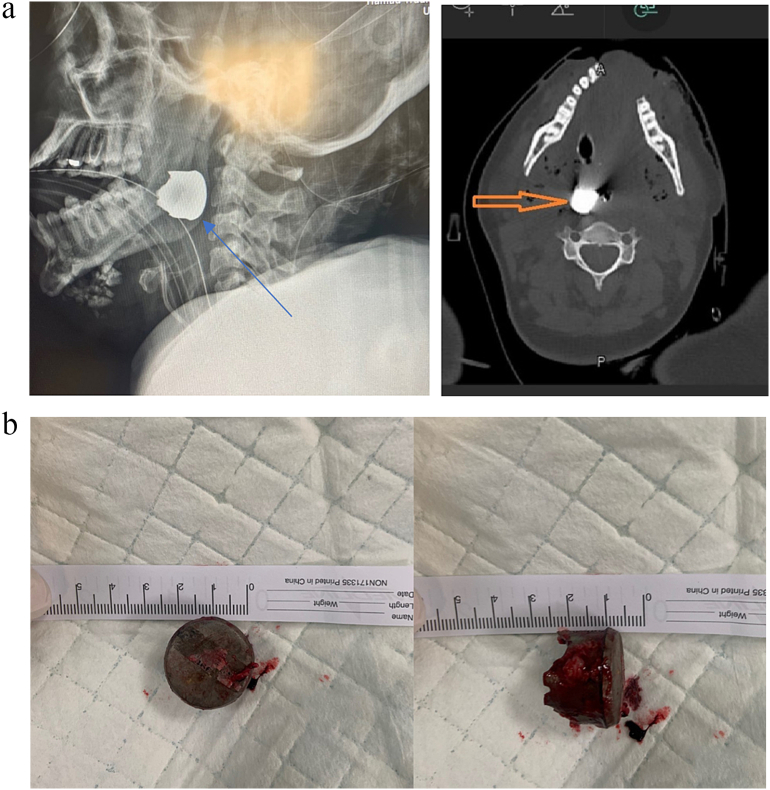
a: Imaging for case 4: arrows show the projectile airbag component. b: The projectile airbag component.

A 37-year-old female unrestrained driver of a 9-year-old compact sedan was injured in a T-bone collision with impact on the driver's side. The airbag was deployed. At scene she was found to be entrapped and in cardiac arrest. She had a prolonged extrication time; CPR was initiated with bilateral needle decompression, and she was revived and transported to the trauma bay. She had a large deformity in the lower face with a split mandible. CT scan showed diffuse cerebral edema, fracture of the basioccipital in the midline extending to the margin of the foramen Magnum with a comminuted displaced fracture of the symphyseal and para symphyseal regions, anterior body of the mandible on the left side and a metallic FB in the palate (Fig. 6a-b). She also had a fracture of the C1 vertebrae. Additionally, she sustained bilateral pneumothoraces with grade II spleen and liver injuries. She showed no signs of brain activity. A rounded metallic object was retrieved via the mouth. She also had post-arrest hypoxic ischemic encephalopathy and expired 6 days post-crash [Table 1].

**Table 1:** Description of the six airbag-related injuries.

Patient	Patient age/sex	Crash details	Vehicle age [year of manufacture]	Airbag injuries	Other [non-airbag] Injuries	Outcome	Seatbelt	Foreign body dimensions	Autopsy or police report	Remarks
1	29/M	Head on collision, driver	13 (2008)	Right mandibular fracture	None	Alive, dis-charged on 14th post-injury day	Yes	22 × 21 × 21 mm	None	The patient underwent ORIF Right mandible
2	43/M	Single vehicle frontal collision, driver, entrapped, extricated by EMS	13 (2009)	Sucking left upper chest wound with active bleeding left rib and sternal fracture, pulmonary contusion	None	Expired on 128th post-injury day	Yes	25 × 25 mm Measured on CT images	Not available	The patient underwent emergent Left thoracotomy ARDS needed prolonged ECMO
3	24/M	Head on collision, driver, single vehicle collision	11 (2011)	Right sucking chest wound lung laceration, hemothorax right scapula fracture.	None	Alive, dis-charged on 8th post-injury day	Yes	20 × 15 mm	None	Right thoracotomy foreign body removed via incision on his back
4	37/F	T-bone or side impact on driver's side, driver, entrapped in vehicle, found in cardiac arrest	9 (2013)	Fracture of the C1 vertebrae, basioccipital in the midline extending to the margin of the foramen Magnum with a comminuted displaced fracture of the symphyseal and para symphyseal regions, anterior body of the mandible on the left side	Diffuse cerebral edema due to hypoxic encephalopathy Grade 2 spleen and liver injuries Sternal fracture Bilateral pneumothorax	Expired on 6th post-injury day.	No	20 × 15 mm	1- Compound fracture of the lower mandible with skin loss and fracture of the anterior Right side and posterior parts of the C1 vertebrae 2- Compound fracture of the base of the skull reaching the foramen magnum 3- Compound fracture Left mandibular joint 4- These injuries are irrefutably caused by a foreign body, measuring 2 cm, with sharp edges, this foreign body was not extracted by the doctors because of its location and the patient was declared brain dead 5- Injuries that can't be explained by the FORIEGN BODY: sternal fracture, liver and spleen	No surgical intervention. Declared brain dead on day 5
5	28/M	Mechanic working on airbag No damage to vehicle	11 (2009)	Right mandible fracture, penetrating injury to Right neck small Right parietal sub arachnoid hemorrhage	None	Alive, dis-charged on 7th post-injury day	Not applicable	None found	None	Debridement and suturing of wounds. He later had an elective mandible fixation
6	21/M	Sedan vs. truck driver	11 (2008)	Puncture wound on his chest wall, Right clavicle fracture	None	Alive, dis-charged on 4th post-injury day	Yes	2.5 × 2.5 × 2.1 cm	None	Wound exploration and retrieval of a metallic foreign body

## Case-5

**Fig. 7:**
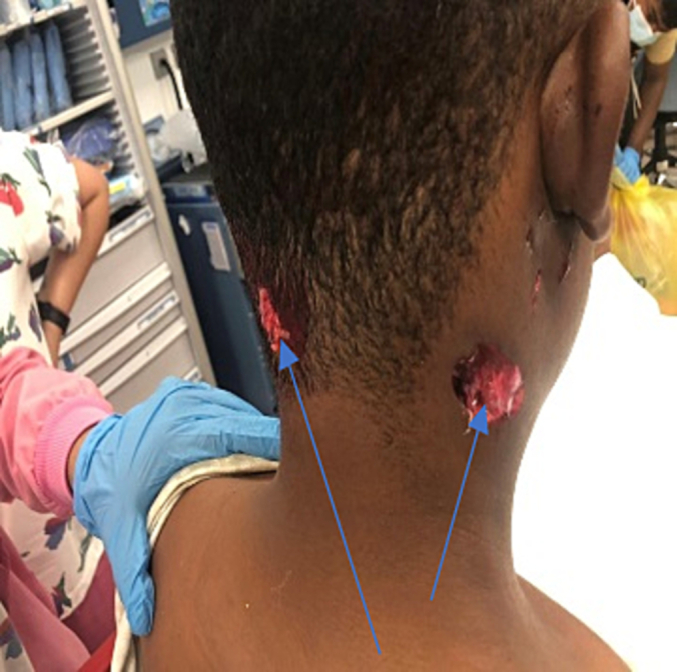
Case 5: through and through and through injury in the back neck.

A 25-year-old male mechanic who was working on airbag of an 11-year-old compact sedan when the airbag deployed suddenly. He was found by EMS personnel outside of the vehicle with a through and through penetrating injury to the right side of the neck (Fig. 7). He was awake and agitated. He remained hemodynamically stable and had a patent airway. CT scan revealed a small parietal subarachnoid hemorrhage and a right sided mandible fracture with no other major traumatic injuries. He underwent debridement and suturing of wounds on his neck and closure of the wound and was observed in the ICU. Later, he underwent elective mandible fixation. Recovery was uneventful with discharge 7 days post-injury, without any neurological deficits.

## Case 6

A 21-year-old male driver of an 11-year-old mid-size sedan was injured in a frontal collision. He was found to have a puncture wound on his chest wall and was hemodynamically stable with a GCS of 15. In the trauma room he had a CXR and a CT scan which revealed a foreign body in the chest wall with no other major injuries. He underwent wound exploration and retrieval of a metallic FB in the OR. His post-operative course was uneventful, and he was discharged 4 days post-injury.

## Discussion

**Fig. 8:**
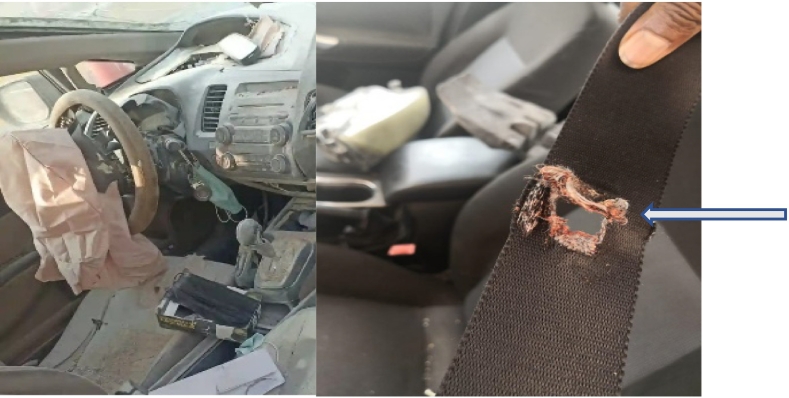
Interior of one of the cars involved in the present case series; the arrow indicates a hole in the seatbelt made by a projectile component.

Over the last three decades, airbags have revolutionized motor vehicle safety and have made a significant impact in injury prevention [3]. However, it has not been proven to be a foolproof system. Given the significant force created by their deployment, various patterns of injury have been described with several reports of deaths [2,4,5]. The impact of a blunt trauma force can produce injuries to the head and neck including, facial fractures, cervical spine injuries, orbital injuries, burns as well as torso injuries such as rib fractures, pneumothorax, aortic injuries [2,5,6]. However, in the absence of penetrating parts of the airbag, this kind of injury is difficult to explain and describe. We reviewed six cases over 3 years and presented a unique pattern of penetrating injuries caused by airbag malfunction resulting in the deployment of a metallic fragment of the inflator component as a penetrating projectile material (Fig. 6b). This kind of projectile component was reported in casualties which resulted in large scale recalls of vehicles in 2015 due to defective inflators from a single manufacturer [4]. Two out of six patients died in our case series. Fig. 8 is an example of one of the crashed cars and showed the penetration of the seatbelt by the projectile component. Although, the vehicle' s recall for airbag safety discussion is important, it is beyond the scpoe of this paper . Vehicles in this series were at least 9 years old or more, at the time of the crash. The age of a vehicle could be a risk factor for malfunctions of vehicle's airbag. Moreover, it is possible that the long-term high ambient environmental humidity and temperature (as in our region for example) might have a contribution to airbag deployments over time making them prone to unexpectedly explode [7]. Other factors might also make the airbag propellant of vehicles more liable to degradation. In a collision, where the airbag is triggered, there's a risk the inflator will rupture under too much internal pressure, sending metal shrapnel shooting into the cabin.

**Fig. 9:**
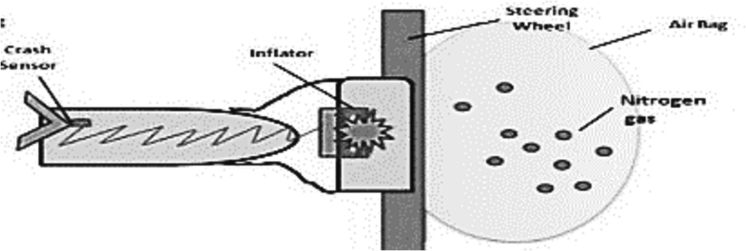
Components of airbag (source: https://www.popsci.com/how-airbags-are-supposed-to-work/ accessed on 06 November 2022).

Airbags consist of six parts (Fig. 9); an accelerometer which detects sudden change in the speed of the car and any impacts. The circuit is triggered by the accelerometer after any impact which then activates the heating element resulting in activation of the explosive charge which rapidly inflates the nylon airbag with nitrogen gas at 200 miles per hour [4]. The time taken between crash detection and complete deployment is approximately 0.05 s. This rapid force ,though usually lifesaving, has the potential to cause catastrophic damage.

Airbag inflator malfunctions can result in deployment of metallic projectiles at high speeds like firearms causing devastating injuries and even death as described in our patients and others [4,[8], [9], [10]]. We lack proper documentation for the actual rate of airbag deployment. Previously, we studied 1830 MVC trauma patients over 3 years and we found, in the HTC registry, 45 cases with documented airbag deployment (2.5 %), moreover, data on the airbag-related injuries were not documented previously in our database [11].

While injuries due to airbag deployment are uncommon or underreported, this report does not reflect the actual incidence of airbag-related injuries in the country. All injuries were caused by a metal fragment from the inflator component which acted as a projectile causing penetrating injury. Although these findings may not have direct clinical impact in terms of management of patients it should bring attention to injury prevention teams as well as state and industrial actors to reevaluate safety standards in vehicle manufacturing. Recently, technology advances have changed the force that the airbag deploys, and the size and shape of airbag, these are expected to improve airbag safety and benefits [12].

## Limitations

As these data were collected retrospectively (2019–2022), some data were not available. The car VIN (vehicle identification number), make and model were available for five cars, however, there is no traffic or institutional agreement to disclose such information to a third party. Therefore, we cannot assume the exact company that produced these airbags in this seris as it is out of our paper scope and permission. We are already working with the pertinent government agencies who will be conducting the full investigations of these injuries and applying the attendant product safety standards. The autopsy was available for one of the two victims (Table 1). The fatalities cannot be directly linked to the air bag rupture alone, however the airbag projectile components were found at the site of body injury of all cases as well as the autopsy of a deceased case. Moreover, we cannot calculate the real incidence of injuries and fatalities from this small series. Police accident report is also not to be shared without agreement and permission from the concerned authorities.

## Conclusion

This case series would help the trauma healthcare providers to better understand airbag-related injuries which could influence the management of road traffic injury patient who has an associated penetrating trauma. Also, it would bring attention to injury prevention teams as well as the state and industrial authorities to reevaluate safety standards in vehicles. Sharing this information with local authorities who govern product safety standards and recalls is essential to ensure that more actions are taken to improve the public safety in this regard.

## Ethical approval

The Medical Research Center of Hamad Medical Corporation has granted permission for this case report to be published on condition that no patient-identifiable data (including patient name and photograph) are included (IRB# MRC-04-22-719). No informed consent was needed as data were collected anonymously with not direct contact.

## Funding

None.

## CRediT authorship contribution statement

All authors have made a substantial contribution to the concept, design of the work; and interpretation of data, drafted the article and revised it critically for important intellectual content, and approved the version to be published.

## Declaration of competing interest

The authors declare no conflict of interest.

## Data Availability

All data were included in the manuscript, table and figures.

## References

[1] WHO (March 19 2021). Injuries and violence. https://www.who.int/news-room/fact-sheets/detail/injuries-and-violence#:~:text=Across%20all%20ages%2C%20the%20three,risk%20of%20injury%20and%20violence.

[2] Wallis L.A., Greaves I. (2002). Injuries associated with airbag deployment. Emerg. Med. J..

[3] Newer cars are safer cars. NHTSA (n.d.). Retrieved October 11, 2022, from https://www.nhtsa.gov/newer-cars-are-safer-cars.

[4] (2021, April 26). Pop. Sci..

[5] Mohamed A.A., Banerjee A. (Aug 1998). Patterns of injury associated with automobile airbag use. Postgrad. Med. J..

[6] Ozkan B., Akinci K., Uysal C.A., Ertas N.M. (2021). Bilateral isolated blowout fracture due to airbag deployment. Turk. J. Plast. Surg..

[7] https://www.nytimes.com/2016/05/05/business/takata-airbag-defect-recall.html/.

[8] Air Bags. (n.d.). NHTSA. Retrieved November 3, 2022, from https://www.nhtsa.gov/equipment/air-bags.

[9] Jothee S., Shafie M.S., Mohd Nor F. (Oct 2018). Fatal penetrating neck injury due to defective airbag inflator. Forensic Sci. Int..

[10] Usumoto Y., Hikiji W., Kudo K., Tsuji A., Ikeda N. (Nov 2008). An unusual case of fatal airbag injury. Fukuoka Igaku Zasshi.

[11] El-Menyar A., Consunji R., Asim M., Abdelrahman H., Zarour A., Parchani A., Peralta R., Al-Thani H. (2016). Underutilization of occupant restraint systems in motor vehicle injury crashes: a quantitative analysis from Qatar. Traffic Inj Prev..

[12] https://www.edmunds.com/car-safety/the-evolution-of-front-airbags.html#:~:text=Advanced%20Airbags,or%20%22multi%2Dstage.%22/.

